# Why TDP-43? Why Not? Mechanisms of Metabolic Dysfunction in Amyotrophic Lateral Sclerosis

**DOI:** 10.1177/2633105520957302

**Published:** 2020-09-17

**Authors:** Mara-Luciana Floare, Scott P. Allen

**Affiliations:** Sheffield Institute for Translational Neuroscience (SITraN), University of Sheffield, Sheffield, UK

**Keywords:** TDP-43, SOD1, FUS, C9orf72, ALS, metabolism, metabolic defects, glycolysis, mitochondria, fatty acids oxidation

## Abstract

Amyotrophic lateral sclerosis (ALS) is a rapidly progressive and fatal neurodegenerative disorder for which there is no effective curative treatment available and minimal palliative care. Mutations in the gene encoding the TAR DNA-binding protein 43 (TDP-43) are a well-recognized genetic cause of ALS, and an imbalance in energy homeostasis correlates closely to disease susceptibility and progression. Considering previous research supporting a plethora of downstream cellular impairments originating in the histopathological signature of TDP-43, and the solid evidence around metabolic dysfunction in ALS, a causal association between TDP-43 pathology and metabolic dysfunction cannot be ruled out. Here we discuss how TDP-43 contributes on a molecular level to these impairments in energy homeostasis, and whether the protein’s pathological effects on cellular metabolism differ from those of other genetic risk factors associated with ALS such as superoxide dismutase 1 (SOD1), chromosome 9 open reading frame 72 (C9orf72) and fused in sarcoma (FUS).

## Introduction

Amyotrophic lateral sclerosis (ALS) is a multi-pathogenic, late-onset disorder, characterized by selective loss of upper motor neurons in the brain, and lower motor neurons in the brainstem and the spinal cord.^1,2^ Although ALS is a fatal disease which significantly impacts on the quality of life of those affected, currently there is no curative treatment available and the present therapeutic strategies offer minimal palliative care. As a result, there is an urgent need for developing treatments that could prevent the progression of the disease and compensate for its detrimental effects.

Several genetic causes have been identified as central to the pathogenesis of ALS, one of increasing recognition being the TAR DNA-binding protein 43 (TDP-43).^3-7^ Moreover, a growing body of evidence indicates that an imbalance in energy homeostasis is a highly prevalent aspect of ALS pathogenesis, which correlates closely to disease susceptibility and progression.^1,8^ Considering the recognition of TDP-43 as one of the histopathological signatures of ALS, and prevailing evidence of metabolic dysfunction as a ubiquitous aspect of the disease, it would not be conjectural to think that TDP-43 could also have a contribution to this deregulation of metabolic homeostasis.

In this review, we will discuss the latest findings in the field, highlighting the contribution of TDP-43 to mitochondrial defects, changes in energy-generating pathways and altered metabolic profiles in ALS. In addition, we will also discuss how the contribution of TDP-43 pathology to energy imbalance differs to that of other well-known genetic causes of ALS such as the superoxide dismutase (SOD1) gene, chromosome 9 open reading frame 72 (*C9orf72*) gene and fused in sarcoma (FUS). We hope to provide insight into the possible molecular mechanisms underpinning neurodegeneration in ALS and to highlight potential therapeutic targets which could be explored by future research.

## The physiological function of TDP-43 in the central nervous system

Transactive response DNA binding protein 43 (TDP-43) is a highly conserved and ubiquitously expressed member of the heterogeneous nuclear ribonucleoprotein (hnRNP) family, which plays an essential role in RNA metabolism.^9^ The importance of TDP-43 comes from its sequence specific-binding to single-stranded RNA molecules, which confers its ability to control cellular events such as pre-mRNA splicing and repression modulation of gene expression.^3^

**Figure 1.**
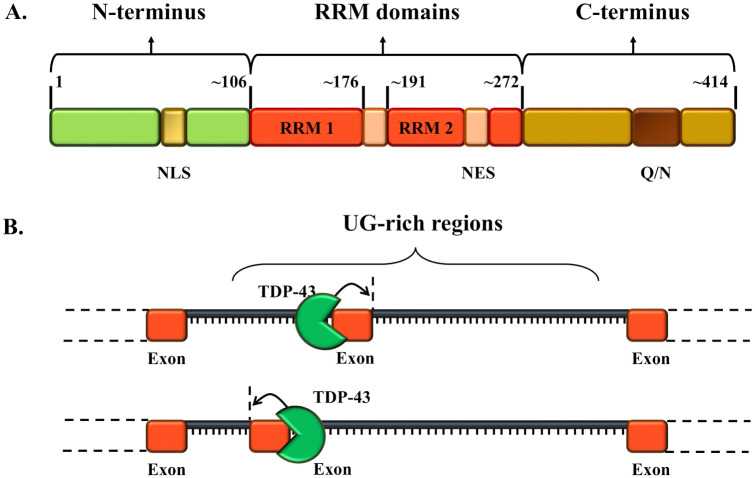
A. Schematic representation of the primary structural architecture of TDP-43 protein. The high functionally relevant regions are annotated (NLS = nuclear localization signal; NES = nuclear export signal; Q/N = core region rich in glutamate/asparagine); RRM = RNA recognition motifs. B. Schematic representation of TDP-43 targets, and the result of TDP-43 binding to UG-rich sequences on mRNA. After TDP-43 comes in close localization to the UG-rich sequences (GUGUG and UGUGU) on mRNAs, an intracellular signaling cascade indicates that the pre-messenger RNA requires additional processing and transport to the cytoplasm post-splicing.

TDP-43 is encoded by the TARDBP gene, located on chromosome 1, and is composed of 414 amino acids which possess a complex structural organization. Even though the 3D structure of TDP-43 has not been fully characterized yet, its holistic structure is thought to recapitulate the classical domains of hnRNP proteins: an N-terminal region, two RNA recognition motifs (RRM1 and RRM2), and a C-terminal domain (Figure 1a). The structural organization of the TDP-43 subunits allows the protein to participate in stress granule formation, and to interact with various binding partners in order to modulate its own splicing and self-organization events.^3,9^ Moreover, the two RRMs regions possess a very high affinity for UG-rich sequences, which enables TDP-43 to control the maturation of pre-mRNAs and define its splicing patterns^10^ (Figure 1b).

The highly specific interaction of TDP-43 with RNA molecules depends on a sequence of cellular events and signaling cascades which are initiated when TDP-43 comes into proximity of 5ʹ or 3ʹ splice sites of long introns, untranslated regions or several exons. This co-localization allows the binding of TDP-43 to UG-rich sequences, further signaling that the pre-messenger RNA requires additional processing and transport to the cytoplasm post-splicing. Besides splicing modulation, TDP-43 controls mRNA life cycle, formation of ribonucleoprotein granules and regulation of non-coding RNAs.^4^ In addition to RNA binding, TDP-43 also binds DNA.^11-17^ Moreover, recent work supports the involvement of TDP-43 in the nonhomologous end joining (NHEJ)-mediated DNA double-strand break (DSB) repair pathway.^18^ TDP-43 rapidly interacts with NHEJ and DNA damage response factors to facilitate the restoration of chromosome breaks, whereas its experimental depletion significantly alters NHEJ repair response and induces the accumulation of genomic DNA DSBs.^18^ Crucially, in the context of neurodegeneration TDP-43 has been suggested to possess an elevated pathogenic potential, due to its highly dynamic and flexible C-terminal.^3^ The C-terminal region of TDP-43 has an intrinsically disordered organization, which makes it highly prone to aggregation^19^ and promotes toxicity.^20,21^ In addition, TDP-43’s C-terminal compartment also presents a highly unstable helix-turn-helix region,^22,23^ which, upon translation, can facilitate the formation of amyloid-like fibrils with prion-like infectious properties.^22,24,25^ Moreover, the C-terminal fragment of TDP-43 can also promote the formation of dynamic protein droplets, which have been suggested to play an important role in the generation of stress granules.^26^

## Amyotrophic lateral sclerosis, a member of the motor neuron disease spectrum

**Figure 2.**
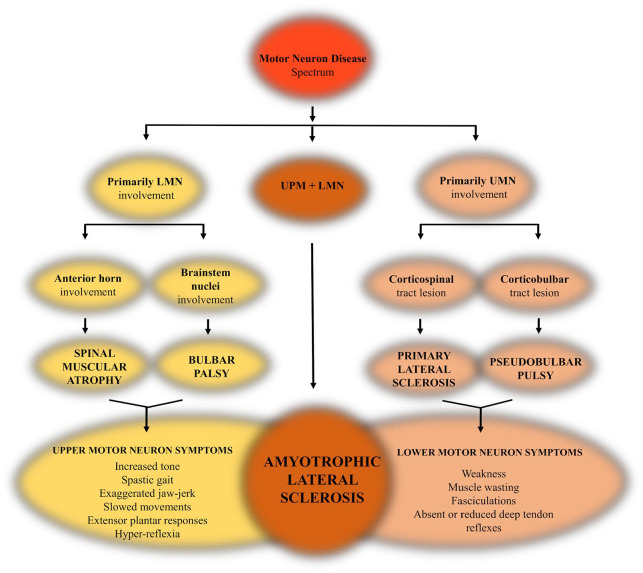
Schematic representation of the motor neuron disease spectrum and its accompanying symptomatology.

Motor neuron diseases (MNDs) recapitulate a group of complex, neurodegenerative conditions which affect the nervous system, leading to progressive death of motor neurons, with amyotrophic lateral sclerosis (ALS) being the most common form (Figure 2). ALS is a late-onset neurodegenerative disorder, characterized by selective loss of upper motor neurons in the motor cortex which control bulbar muscles, and lower motor neurons in the spinal cord which control skeletal muscles.^1^ This sustained neuronal deterioration leads to a series of motor dysfunctions such as difficulty in walking, limb weakness, slurred speech, swallowing problems, impaired respiratory function. Death typically occurs 2 to 5 years post diagnosis.^27^ Due to the fast nature of progression and low survival rate, ALS is regarded as a disorder of low penetrance and reduced incidence.^2^ However, this incurable condition is far from infrequent, with the latest data indicating a 1 in 300 to 400 lifetime risk factor, which continuously increases with life progression.^28^

**Figure 3.**
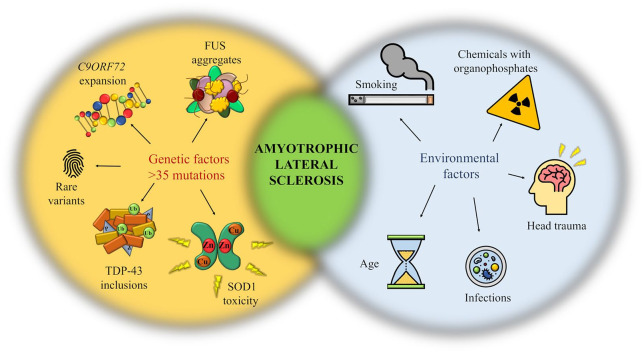
Schematic representation highlighting the genetic and environmental factors associated with ALS. ALS is a multistep process in which genetic mutations, rare variants and a plethora of environmental factors work together toward developing the condition, the last exposure being considered the disease trigger.^29^

In terms of its causation, the great majority of disease manifestation are considered sporadic in nature (90% of all cases). However, more than 35 different genes have been linked to ALS, with the rate of gene discovery showing a quadrennial twofold increase.^2^ Large-scale genome-wide association studies have identified four main genes as highly frequent ALS causative factors: the superoxide dismutase (SOD1) gene, TAR DNA binding protein (TARDBP) gene, chromosome 9 open reading frame 72 (*C9orf72*) gene and fused in sarcoma (FUS) (Figure 3).

Currently, there are limited therapeutic strategies for ALS patients, but alternative approaches such as antisense oligonucleotides (ASOs) treatments are emerging as a viable treatment in SOD1-ALS cases. A recent study indicated that SOD1-ALS patients who received tofersen, an ASO which controls the degradation of SOD1 messenger RNA, measured a dose-dependent reduction from baseline in the total CSF SOD1 concentration of up to 36% after 12 weeks of treatment.^30^ Even though the safety and efficacy of tofersen are currently under investigation, the aforementioned study provides evidence that precision-medicine approaches using ASO could open the way to therapeutic interventions in ALS and potentially other neurodegenerative conditions.

## Mislocalization of TDP-43, a critical aspect of ALS pathogenesis

**Figure 4.**
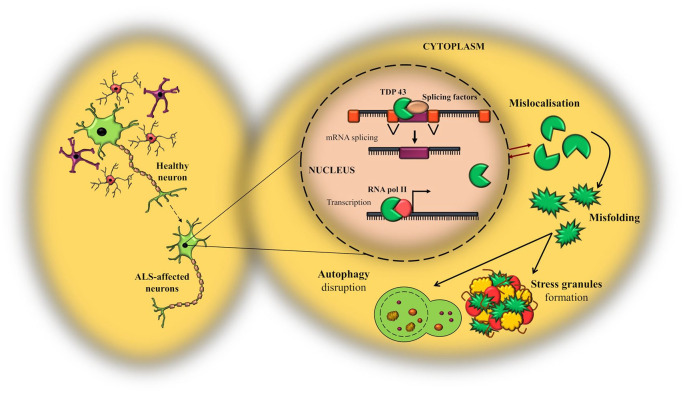
Schematic representation of TDP-43 cytoplasmic mislocalization, and the consequential cascade of pathological molecular events. In a physiological state, TDP-43 resides primarily in the nucleus, where it regulates major aspects of RNA metabolism. In a pathological context, defects in the structural components of the import/export machinery can facilitate the mislocalization of TDP-43 to the cytoplasmic compartment followed by its accumulation in cytoplasmic inclusions. The consequential abnormal signaling of TDP-43 promotes the formation of stress granules and impairs important pathways for cellular degradation such as autophagy. RNA pol II, RNA polymerase II.

Great scientific effort has been invested into characterizing the pathogenesis of ALS, but many of the cellular mechanisms underpinning disease progression remain unclear. However, it is well recognized that the aggregation of hyper-phosphorylated and ubiquitinated TDP-43 protein into cytoplasmic inclusions represents one of the primary neuropathological hallmarks of ALS, supporting the contribution of TDP-43 pathology to neurodegeneration.^3,4^ In a physiological state, TDP-43 is mainly localized in the nucleus, where it controls gene expression and pre-mRNA splicing, and modulates the regulation of its own mRNA, as discussed above. However, a small proportion continuously shuttles to the cytoplasm to perform some of its other functions, including the control of mRNA stability and translation.^3^ This dynamic character of TDP-43 is dependent on distinct protein import systems, and defects in the import/export machinery have been suggested to represent one of the causative steps in TDP-43 pathology^3,4^ (Figure 4). Multiple hypotheses have been put forward to explain the underlying pathogenic mechanisms of action of TDP-43, leading to a debate as whether TDP-43 pathology stems from a loss of nuclear function, a gain of toxic cytoplasmic activity, or a dominant-negative mechanism.^31^ A context in which loss of function (haploinsufficiency) is the primary pathological modulator of TDP-43 activity would imply that the mutant gene has either a reduced function or no function at all, but does not interfere with the signaling of the wild-type allele. Reducing the expression of the mutant gene will reproduce the disease phenotype in a preclinical model, whereas overexpression of the mutant or the wild-type allele will have no pathological outcome. Oppositely, in a gain-of-function scenario, the mutant gene may become hyperactive in one of its normal activities, leading to cellular toxicity. In this framework, overexpressing either the mutant or the wild-type form will eventually recapitulate the disease characteristics. However, it may be also possible for the mutant gene to acquire a novel function which is independent of its physiological signaling, in which case upregulating the mutant gene alone will trigger the disease. Finally, in a dominant-negative mechanism, the mutant allele possesses an inhibitory effect on the wild-type form, downregulating its activity. Even though it is essentially a loss of function, the negative effects extend toward the endogenous wild-type as well, differentiating it from a situation of haploinsufficiency. This scenario can be mimicked in an experimental setting by overexpressing the mutant allele, which is expected to consequently inhibit the endogenous wild-type protein.^31^ In the period immediately following the discovery of TDP-43 as a neuropathological component of ALS, studies were focused on gain-of-function models, providing evidence that upregulation of both the mutant and the wild-type allele can cause neurodegeneration (for an extensive review see^32^). However, with the progression of research, the paradigm has shifted toward an understanding that the aggregation of TDP-43 into cytoplasmic inclusions confers both a loss of nuclear activity, as well as a toxic gain of cytoplasmic function.^33^ Supporting the detrimental effects of TDP-43 cytoplasmic aggregates, a recent study performed proteomic analysis on primary cortical neurons, patients fibroblasts and neurons derived from stem cells, and indicated that cytoplasmic accumulation of TDP-43 is the result of defects in the localisation of nucleoporins and transport factors. This localization defect concomitantly impairs the nuclear pore complex, causing nucleocytoplasmic transport abnormalities.^34^ Interestingly, experiments on neurons from post-mortem ALS cases that did not exhibit nuclear TDP-43 expression indicated that loss of TDP-43 function results in nucleocytoplasmic transport impairment, RNA processing defects, DNA damage and altered histone processing.^35^ Based on the previous belief that the nuclear depletion of TDP-43 precedes the formation of cytoplasmic inclusions,^31^ it may be possible that loss of nuclear TDP-43 contributes to neurodegeneration via a feedback forward mechanism in which cytoplasmic mislocalization and aggregation is a secondary insult which additionally elevates cellular toxicity. Baskaran et al. (2018) took this concept further when they suggested that TDP-43 mislocalization led to neuronal loss via a disruption of cytoskeletal integrity in cortical neurons, which eventually impaired axonal growth and microtubules dynamics.^36^

Nevertheless, even though extensive research has been focusing on the loss versus gain of function debate, valuable evidence proposes additional pathogenic mechanisms. For example, Wils et al. (2010) showed that transgenic models of TDP-43 developed significant neuronal cytoplasmic inclusions, mainly composed of phosphorylated and ubiquitinated TDP-43. Yet, the prevalence of aggregates in which TDP-43 was not the main protein deposited led to the suggestion that cytoplasmic accumulation of TDP-43 is not a requisite for TDP-43-mediated toxicity in ALS, and that TDP-43 might exert a destructive function by impairing protein degradation pathways.^37^ This hypothesis is in line with previous research highlighting the contribution of TDP-43 to modulating autophagy, a crucial self-degradation process of cleaning out defective cells and organelles to allow the regeneration of newer, healthier cellular components.^38^ In this context, it may be possible that abnormalities in the physiological functioning of TDP-43 lead to downstream defects in the autophagy pathway, initiating a positive feedback loop which enhances the aggregation of cytoplasmic inclusions (Figure 4). Indeed, genetic engineering of autophagy modulators such as Atg5 or Atg8 initiated neuronal defects and resulted in neurodegeneration,^39^ whereas more recent studies revealed that mutations in several genes responsible for autophagy could be classified as etiological factors for ALS regulation.^40^ Interestingly though, pharmacological manipulation of autophagy resulted in conflicting results,^41,42^ where factors such as differences in methodologies, the nature of the drug tested or the disease stage at which the drug administration began lead to significant variability.

When discussing the possible contribution of TDP-43 to neuronal toxicity, it is important to note its involvement in the formation of stress granules (SGs). As mentioned above, low levels of TDP-43 are continuously shuttled to the cytoplasmic compartment to control mRNA stability. At this level, when exposed to various environmental stressors such as viral infections, heat shocks, osmotic or oxidative agents, TDP-43 can be recruited to cytoplasmic RNA granules called stress granules. These stress granules are membrane-less organelles which capture non-essential mRNAs, RNA-binding proteins or translation factors, and hinder the translation of mRNA targets to ensure cell survival and to protect them against degradation due to cellular insults.^43,44^ This property is conferred by the C-terminus head of the TDP-43 protein, which enables liquid-liquid phase separation (LLPS) activity. Normally, the process of SG formation is reversible and SGs get immediately absorbed after the stress has passed. However, certain ALS-related TDP-43 mutations have been suggested to act on the LLPS properties, impairing SG dynamics and facilitating the accumulation of TDP-43 inclusions (Figure 4).^4,44^

The studies discussed in the section above provide evidence for the contribution of TDP-43 dysfunction to ALS pathogenesis, arguing that TDP-43 pathology reflects a complex interplay between loss and gain of function mechanisms which alters neuronal health and eventually promotes cell death. However, with the contemporary research in mind, the question of whether the cytoplasmic mislocalization and accumulation of TDP-43 are a cause or a consequence of ALS remains to be addressed in future studies for a comprehensive, in depth understanding of disease etiology and progression.

## The pathogenic contribution of fused in sarcoma protein to ALS

Fused in sarcoma is another member of the RNA binding proteins group, whose physiology and cellular activity show great synonymy to those of TDP-43, both in a healthy and a diseased state.^45^ Similar to TDP-43, FUS’ primary localization is within the nucleus, and its physiological activity has implications on different features of RNA metabolism, including transcription and splicing regulation,^46,47^ mRNA transport^48,49^ and DNA damage repair.^50,51^ Moreover, a percentage of the protein is present in the cytoplasm and the neuronal processes, where it carries out functions associated with mRNA transport and local protein translation.^52^ Considering the dynamic nature of the protein, defects in the nuclear import machinery could have a detrimental effect on both the nuclear function and the cytoplasmic activity. Indeed, retention of FUS in the cytoplasm, followed by its accumulation in cytoplasmic inclusion, is a well-recognized pathological feature of ALS.^53^ However, as for TDP-43, there is a continuous debate around the contribution of the associated reduction in nuclear expression or cytoplasmic gain of toxic function to motor neuron death in ALS pathogenesis. As nuclear FUS plays a crucial role in modulating RNA metabolism, mutations associated with its coding gene could hinder genetic expression and impair alternative splicing events, leading to transcriptome deterioration as suggested by FUS knock-out models.^45^ However, complete inactivation of FUS resulted in post-natal mouse death, making it difficult to identify any neuro-related phenotypes.^54^ A more substantiated hypothesis proposes cytoplasmic mislocalization and gain of toxic attributes as the initial steps in the disease cascade, arguing that reduced nuclear activity does not directly contribute to motor neuron degeneration in ALS.^55^ Experimental efforts have been made to compare the pathological phenotype of models expressing mutant cytoplasmic FUS against those with complete ablation of FUS protein. The results showed perinatal loss of motor neurons in the knock-in group, but not in the knockout cohort, and are substantiated by the observation that rescue of nuclear FUS arrested motor neuron death.^55^ Moreover, the accumulation of FUS cytoplasmic inclusions has been associated with the formation of SGs,^56^ whose pathological traits have been discussed in the context of TDP-43 pathology in the section above. Considering the evidence discussed so far, even though the mechanisms through which FUS contributes to neurodegeneration remain an open question, impairments in FUS functionality is an undisputed feature of ALS pathology and its pathogenicity cannot be contested.

## Superoxide dismutase 1 enzyme pathology in ALS

Pathological expression of SOD1 enzyme has been described as the first genetic cause of ALS, being responsible for one fifth of familial disease cases.^57^ SOD1 is a highly conserved and ubiquitously expressed protein, typically localized to the cytoplasm and more sparsely in the nucleus, lysosomes and mitochondrial intermembrane space.^58^ As a requisite cytoplasmic antioxidant, SOD1’s primary function is to catalyze the conversion of highly toxic superoxide radicals (oxygen molecules which possess an additional electron) into molecular oxygen and hydrogen peroxide, providing, as a result, a defence mechanism against ROS-driven stress.^59^ However, SOD1 activity extends to other roles independent of its dismutase-like character, including respiration repression and energy metabolism regulation,^60^ protein nitration,^61^ copper buffering,^62^ immunomodulation,^63^ zinc homeostasis,^64^ and phosphate activation.^65^ Initially, SOD1 pathology was linked to neurodegeneration in ALS via a loss of physiological function and a consequent accumulation of toxic superoxide radicals. However, subsequent research on transgenic models of ALS overexpressing both the endogenous mouse gene, as well as the mutant human SOD1, contradicted the incipient theories, showing that the majority of these disease models exhibit enhanced dismutase activity and progress into adult-onset motor deficits with distinctive loss of lower motor neurons.^66^ As a result, a new hypothesis was put forward, arguing that SOD1 pathology is not associated with a reduced dismutase activity, but with the acquisition of one or more toxic properties of the mutant protein.^67^ In this context, SOD1 has been suggested to contribute to neuronal death by impairing the expression of the glutamate transporter EAAT2, leading, as a result, to accumulation of glutamate at the level of the synaptic cleft and consequent glutamate excitotoxicity.^68^ This proposed mechanism was supported by evidence from SOD1 mutant mice, which exhibited a 50% reduction in the level of EAAT2 protein in the spinal cord,^69^ and a further complete depletion in EAAT2 concentration by the end-stage of the disease.^70^ Moreover, EAAT2 expression was reported to be decreased or abnormal in ALS patients as well,^71^ further confirming SOD1 pathogenicity and its contribution to disease progression. Defects in axonal organization and disrupted transport have been suggested to be another effect of SOD1 toxicity that could potentially lead to motor neurons death in ALS.^72^ Moreover, by interfering with the axonal transport machinery, SOD1 toxicity may indirectly impair the distribution of mitochondria to the periphery or impede retrograde delivery of trophic factors.^73^

The findings discussed above are a few of the proposed mechanisms through which SOD1 gain of toxic function could progress to neurodegeneration, complementing the suggestion that loss of dismutase activity is not the driving factor of SOD1 pathology in ALS. Moreover, recent research showed that SOD1 translocates to the nuclear compartment in a sub-group of sporadic ALS patients, and suggested a potential protective function at this cellular level.^74^ The possibility that, once relocated to the nucleus, SOD1 may act as a co-transcriptional factor of DNA repair genes to reduce genomic damage was proposed by experiments which correlated the presence of nuclear SOD1 with significantly increased disease duration.^74^Therefore, in the light of the evidence discussed above, it could be argued that loss of SOD1 dismutase activity possesses a modifying role in ALS pathogenesis, and its potential protective nuclear function could open the way to notable therapeutic strategies.

## C9orf72-mediated pathology in ALS

Understanding the etiopathogenesis of ALS increased significantly in 2011, when the discovery of a hexanucleotide repeat expansion GGGGCC in the *C9orf72* gene was attributed to as much as 33% of all familial cases of ALS and 5% of idiopathic disease forms.^75^ In a non-pathogenic state, the *C9orf72* gene incorporates 5 to 30 hexanucleotide repeats, showing a neurologically healthy phenotype.^76^ As a result, an arbitrary 30-repeats cut-off has been suggested to mark the threshold beyond which the repeat length becomes pathogenic, and expansions of hundreds to thousands of repeats are a highly common genetic feature of *C9orf72*-ALS (C9-ALS) patients.^77^ However, repeat lengths of 30 or more have also been reported in 0.17% of healthy individuals, indicating that the knowledge of the repeat cut-off must be cautiously used both clinically and experimentally.^76^

The physiological function of the *C9orf72* gene in the CNS is still poorly understood, being mostly substantiated by experiments investigating its pathogenic activity. The non-coding expanded repeats have been shown to contribute to *C9orf72* gene silencing in blood samples from C9-ALS patients,^78^ leading to a consequential reduction by almost 50% in the *C9orf72* mRNA and protein levels.^79^ As a result, loss of function mechanisms has been proposed as a pathogenic contribution of *C9orf72* protein to ALS etiology.^80^ So far, bioinformatic analysis has revealed a protein coding domain in the *C9orf72* gene, which shows a direct interaction with the Rab family of GTP-ases.^81^ As Rab proteins are active regulators of many membrane trafficking steps across a wide range of cell types, *C9orf72* loss of function may have downstream effects on protein transport mechanisms. Interestingly, both TDP-43 pathology and TDP-43-negative but ubiquitin-positive inclusions have been often reported in C9-ALS patients, further indicating defects in transmembrane transport and protein degradation in carriers of *C9orf72* repeat expansions.^82^

Interestingly, the possibility that the G4C2 repeat expansions might be pathogenic through a gain of toxic activity emerged when experiments showed that the hexanucleotide extensions can be transcribed into repetitive RNAs in a bidirectional fashion, leading to the formation of nuclear RNA foci.^83^ Even though these repetitive RNAs are located in a noncoding region of the *C9orf72* gene, they can be translated to five different and highly toxic dipeptide repeat proteins (DRPs).^83^ As a result, two scenarios have been put forward, indicating that the expanded repeats could cause neuronal damage either through a toxic gain of function from the RNA repeats, or a destructive signaling of the DRPs.^76,77^ In vivo experiments suggested that the RNA repeat foci co-localize with TDP-43 and FUS, facilitating their sequestration and contribution to dysfunctional RNA-metabolism mechanisms.^84^ Moreover, the G-rich repeats have been suggested to assemble into helical G-quadruplex secondary structure, determining abnormal cellular activity and interactions with other proteins.^85^ In line with these findings, Walker et al. (2017) showed that both RNA-repeat expansions and poly-GA DPRs contribute to the formation of DNA-RNA hybrids called R-loops in *C9orf72* rat neurons, human cells, as well as in spinal cord tissue from C9-ALS patients. The resultant disruption of ATM-mediated DNA repair signaling and accompanying accumulation of DNA breaks appeared to be the result of *C9orf72*-mediated p62 aggregation.^86^ Furthermore, numerous previous studies in cell lines and mouse models suggested that the expression of each individual DRP may be sufficient to cause neurodegeneration through induced nucleocytoplasmic transport defects,^87^ abnormal proteasomal degradation and protein sequestration,^88^ nucleolar stress^89^ or protein translation inhibition.^90^ Nevertheless, these pre-clinical studies recapitulate an overexpressed DRP pathology, aspect that makes it difficult to correlate the results with the endogenous patterns noticed in patients, and which emphasizes the crucial need for more clinical and experimental work.

However, despite the lack of knowledge around the specific molecular pathways downstream to loss of functional *C9orf72*, or gain of toxic *C9orf72* driven activity, both mechanisms are, presumably, contributing to ALS pathogenesis to some extent, and the evidence discussed above supports the existence of complex and profound implications of *C9orf72* pathology on cellular homeostasis and neuronal integrity.

## ALS, a multi-systematic disorder: implications for metabolic dysfunction

An increasingly recognized aspect of ALS pathology is represented by a disturbance in energy balance, which, in the last decades, has changed the experimental approach toward ALS pathogenesis and led to revolutionary insights into disease mechanisms. It appears that ALS patients often report a decrease in energy uptake, which, controversially, is accompanied by an increase in energy expenditure, along with lower body mass index (BMI) in the pre-symptomatic phase of the disease and hypolipidemia at later disease stages.^1^ These changes lead to a state of hypermetabolism, that is considered to be the result of increased respiratory muscle expenditure, and which seems to correlate closely with disease susceptibility and progression. Moreover, this apparently abnormal metabolic condition is correlated with accentuated cognitive impairment and significantly reduced survival rate.^8^ Two recent clinical studies suggested that the risk for ALS was accurately predicted by the reduction in BMI, whereas high fat mass levels prolonged survival.^91^ However, it is important to note the limitations of case-control studies, whose methodology is based on questionnaires regarding the dietary habits of patients before the onset of the clinical manifestations, making such experiments prone to recall bias.

In line with the protective association between vascular risk factors such as high BMI,^92^ hyperlipidaemia^93^ or increased cholesterol levels^94^ and ALS survival,^95^ diabetes appears to be another chronic metabolic disorder involved in ALS etiology. Recent Danish and Swedish large case-control studies suggested a 0.61 to 0.66 odds ratio of diabetes in ALS patients pre-diagnosis, with age related alterations found indicating a protective associations with type 2 but not type 1 diabetes.^96,97^ On average, patients with type 2 diabetes report abnormal energy metabolism with increased levels of lipids in the blood and elevated BMI,^98^ aspects that could explain a possible positive effect of diabetes on ALS. However, even though there is a clear crosstalk between insulin signaling and the underlying mechanisms of ALS,^1^ the direction of this association still needs to be elucidated as, so far, the experimental results are rather contradictory and inconsistent. Recent studies investigating systematic metabolic dysfunction in ALS suggested a protective effect of diabetes in patients aged 65 and above, while the younger groups appeared to be at higher risk.^96,97^ Interestingly, a recent Italian nested longitudinal study found a negative correlation between diabetes and ALS in older individuals,^99^ while previous research in an Asian population suggesting elevated ALS risks in the elderly.^100^ No gender correlations have been distinguished in a Taiwan-based study,^100^ contradicting the results of two other East Asian-nested studies which showed that men with type II diabetes recorded an increased risk of developing ALS compared to women of the same race.^101,102^

The contribution of metabolic dysfunction to ALS-associated neurodegeneration is further supported by evidence from dietary intervention experiments, which indicate that elevated glucose or serum lipids levels could compensate for the hypermetabolic syndrome reported in ALS patients.^99^ The risk of developing ALS appeared to be reduced in both animal models and humans following a diet rich in both vegetal and marine ω-3 polyunsaturated fatty acids (PUFAs).^103^ As PUFAs are known to reduce the expression of nuclear factor κB, a higher PUFAs intake may be helpful in reducing inflammation, oxidative stress and glutamate-mediated toxicity, contributing therefore to neuronal survival.^104-107^ Moreover, in obesity and type 2 diabetes, the levels of an important neurotrophic and neuroprotective protein, progranulin, are known to be elevated^108,109^ and previous research showed that progranulin can also work against the toxic effects of TDP-43.^108^

Nevertheless, despite the high incidence of ALS and impaired insulin signaling, the underlying mechanisms of this associated need to be further investigated in detail. Nevertheless, the imbalance in energy metabolism remains an undeniable characteristic of ALS pathology, and understanding how these systemic metabolic defects are causally connected to motor neurons loss could provide great insight into disease pathogenesis and possible therapeutic strategies.

## Mitochondria pathology, a major driver of metabolic dysfunction in ALS

Mitochondria are a major player in ATP production and lipid homeostasis. Impaired activity of the electron transport chain (ETC), disruption in mitochondrial fusion/fission rates, abnormal morphological changes or calcium buffering defects, can lead to reduced ATP production and dysfunctional glucose and lipid metabolism.^110^ Compelling evidence shows that the function of each component of the electron transport chain is altered in sporadic ALS patients^111^ and even though each genetic cause contributes in a variant-specific manner to mitochondrial pathology, there seems to be a certain degree of overlap in terms of which aspect of mitochondrial function or structural component is compromised.

In this context, the specific interaction between different ALS-associated proteins and the mitochondria appears to be crucial to the infliction of mitochondrial damage in ALS pathogenesis.^112^ For example, mutant TDP-43 has been suggested to possess an internal mitochondrial targeting sequence, which facilitates a preferential binding to the mRNAs of the mtDNA-encoded complex 1 subunits ND3 and ND6, impeding their transcription and leading to consequential reduced expression of complex I.^113^ As part of the complex mechanisms of oxidative phosphorylation, ROS is a physiological by-product which, at homeostatic levels, acts as a signaling molecule. However, abnormal functioning of the ETC can result in an excessive ROS production and consequently promote damage to the mtDNA, RNA, proteins and lipids.^114^ Elevated levels of ROS markers and pronounced ROS-associated damage have been extensively reported in sporadic ALS patients,^115^ as well as in transgenic or cellular models of ALS upregulating either TDP-43^116^ or FUS synthesis.^117^ Moreover, recent studies show that both TDP-43 and FUS pathologies alter the ability of mitochondria to maintain inner membrane integrity and disrupt mitochondria-endoplasmic reticulum (ER) signaling by impairing the binding of the endothelial vesicle-associated membrane protein associated-protein B (VAPB) to the mitochondrial associated protein tyrosine phosphatase interacting protein 51 (PTPIP51).^118^ Defects in ER-mitochondria communication may further impair the Ca^2+^ exchange system between the two organelles, reducing calcium uptake and potentially contributing to a series of additional toxic mechanisms, including loss of protein homeostasis and axonal transport defects.^119^

Interestingly, a sequestration pathological mechanism has been proposed in transgenic models of SOD1 as well, as mutant SOD1 builds up in the intermembrane space and forms aggregates which impair the activity of the ETC, especially complexes I and IV.^120,121^ Moreover, previous research on SOD1 models described decreased calcium homeostasis^122^ and an abnormal interaction between mitochondria and the ER,^123^ but a causal link between these two features of ALS pathology is still to be confirmed in SOD1 mutants. Elevated level of ROS have been reported in SOD1 models as well,^124^ and TDP-43, FUS and SOD1 themselves have been suggested to be a target of oxidative damage, which promotes their aggregation and possibly facilitates the progression of pathological cascades through a damaging positive feed-forward loop.^125^

Consistent with the contribution of TDP-43 to impaired calcium homeostasis, abnormal mitochondrial morphology accompanied by its accumulation in motor axons has been previously reported in mice models overexpressing wild-type TDP-43, but not in mutant cohorts.^126^ These contradictory results may account for different pathological mechanisms between wild-type and mutant TDP-43 forms, which will be discussed in detail in the section below. Similarly, mitochondrial clustering was reported in transgenic SOD1 models as well,^127^ when Vande Velde et al. (2008) demonstrated that misfolded SOD1 creates very tight associations with the outer mitochondrial membrane in mutant SOD1 models,^128^ possibly altering its motility along with others physiological properties.^129^ However, the association of SOD1 with the mitochondria was restricted to the spinal cord, suggesting a potential site-specific pathogenic activity.

Considering the high incidence of TDP-43 aggregation in cases of GGGGCC expanded repeats, one could readily speculate that mitochondrial morphology and its ATP synthase machinery are a target of *C9orf72* pathology as well. Accordingly, experiments on iPSC-derived *C9orf72* motor neurons reported increased DNA damage compared to controls, and a preferential binding of DRPs, especially poly(GR), to mitochondrial ribosomal proteins. This toxic effect of DRPs compromised mitochondrial function and led to elevated levels of oxidative stress in an age-dependent manner, possibly explaining the presence of DNA damage.^130^ However, as mentioned in one of the previous sections, the results of such studies are usually harder to interpret and integrate within the current knowledge, as they express high levels of poly(GR), much greater than those recorded in ALS patients. To overcome this limitation and approximate more to the physiological conditions, Choi et al. (2019) developed an inducible mouse model of *C9orf72*-dependent toxicity in which the levels of (GR)_80_ accumulate in a progressive, controlled manner in cortical motor neurons.^131^ The mice showed significant defects in mitochondrial function and morphology from as early as 3 months of age, before any other cognitive or cellular impairments, arguing that mitochondrial dysfunction is a prodromal event in the ALS disease course, and that lower pathogenic levels of diffusible poly(GR) are sufficient to initiate the disease. The poly(GR) showed affinity for ATP5A1 subunit of the mitochondrial ATP synthase, increasing its degradation via the ubiquitin-proteasome pathway and consequently impairing mitochondrial ATP production. As the disease progresses, a full spectrum of ALS pathological characteristics including synaptic dysfunction, neuronal loss, cognitive impairment, microgliosis and DNA damage was measured, which possibly developed as a consequence of impaired mitochondrial activity and energy balance. Interestingly, TDP-43 pathology appeared to be absent in the transgenic cohort, possibly indicating that poly(GR) toxicity is the initiating factor in C9-ALS, and that TDP-43 pathology follows later in the disease pathogenesis, potentially as a consequence of altered protein transport mechanisms and impaired autophagy.^78^ Since recent evidence reinforces the hypothesis that mutant forms of TDP-43 themselves interact with and impair the assembly and function of the oxidative phosphorylation system, causing toxicity and mitochondrial damage^132,133^ we speculate that TDP-43 may function differently on its own, compared to when it is associated with *C9orf72* pathology. Contrarily, it may also be possible that cascades upstream to TDP-43 inclusions are facilitating the progression of neurodegeneration in C9ALS cases, and that TDP-43 aggregation itself is a consequence, rather than a cause of the disease.

**Figure 5.**
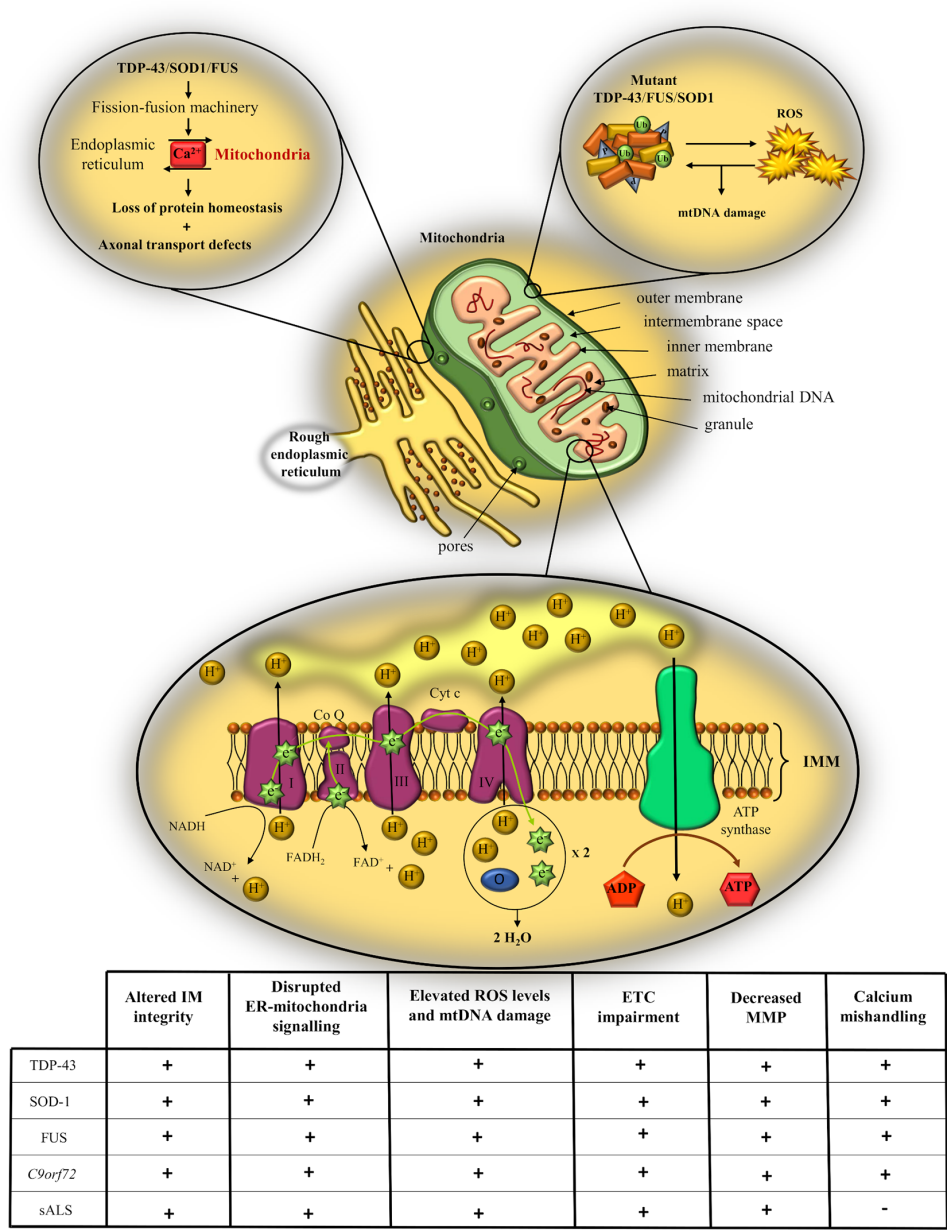
Schematic representation of mitochondrial pathology in ALS. Defects in mitochondrial morphology and physiology can impair the activity of the electron transport chain (ETC), lead to accumulation of reactive oxygen species (ROS) and promote calcium buffering defects. Table summarizing the effects of the main genetic causes of ALS and of the sporadic form on different aspects of mitochondrial structure and functioning. Co Q, coenzyme Q; Cyt c, cytochrome c; ER, endoplasmic reticulum; IMM, inner mitochondrial membrane; MMP, mitochondrial membrane potential; mtDNA, mitochondrial DNA.

Taken together, the studies discussed above support the existence of metabolic dysfunction in ALS, providing evidence for the crucial contribution of mitochondria pathology to ALS pathogenesis (Figure 5). However, even though it is increasingly apparent that multiple aspects of mitochondria morphology and physiology are damaged in ALS, there are still many gaps in our knowledge regarding the molecular pathways involved, gaps that must be filled before shifting our focus toward finding therapeutic strategies against motor neuron degeneration.

## The potential contribution of glycolysis and fatty acid oxidation to restoring energy balance

When discussing energy metabolism and mitochondrial pathology, it is worth taking a step backwards in the pathways of cellular respiration and look more attentively into the possible implications of glycolytic flux and fatty acid oxidation (FAO) on ATP production. Glycolysis is a crucial energy generating process and, at the same time, a vital step for aerobic cellular metabolism which, in its energy-releasing phase, provides the substrates required for oxidative phosphorylation. The general consensus promotes glucose as the principal substrate which satisfies energy requirements in the brain, arguing that, although others organs with increased energy turnover such as the kidney or the heart largely rely on fatty acids oxidation for energy supply, the brain has a preference toward glycolytic rather than oxidative metabolism.^134,135^. The low rate of fatty acid oxidation in the brain might be explained in part by the relatively low permeability of the blood-brain barrier to fatty acids, the higher oxygen demand and superoxide generation for FAO compared to glycolysis, along with the decreased enzymatic capacity for fatty acid degradation.^134^. However, increasing attention has been drawn to lipid metabolism in the central nervous system, since previous studies have suggested that oxidation of fatty acids contributes almost 20% to the total brain’s energy demands,^136^ and caloric-dense diets can improve disease outcomes.^95^ In the light of the current discoveries, there seems to be an exquisite interplay between glycolysis, fatty acid oxidation and oxidative phosphorylation in the brain, and the ability to metabolically switch between these energy-transducing pathways may be crucial in terms of securing the much needed constant energy support to neuronal cells.^137^ In this context, a recent elegant study on a *Drosophila* model of TDP-43-ALS reported the upregulation of key genes involved in glucose metabolism,^138^ reiterating at the same time the contribution of TDP-43 to mitochondrial dysfunction. The results indicate that, as ALS progresses, glycolysis might be upregulated in dying motor neurons as a compensatory mechanism for the loss of mitochondrial ATP, and could even confer neuroprotection by increasing the levels of pyruvate levels, a physiological metabolic intermediate and antioxidant which has been shown to safeguard mitochondria against oxidative stress.^139^ Interestingly, despite the great pathogenic similarity between TDP-43 and FUS and the extensive circumstantial evidence linking both DNA binding proteins to impaired energy metabolism, mutations in FUS appeared to have no effect on glycolytic or mitochondrial energy metabolism in a recent study on iPSC-derived MNs from FUS-linked ALS patients.^140^

Previous work from our laboratory showed that fibroblasts from mutant-SOD1-I113T ALS patients undergo a similar switch in energy generating pathways to that observed in the TDP-43 model.^141^ Our results reported mitochondrial defects and reduced ATP production from oxidative phosphorylation, which, however, was accompanied by an increase in glycolytic flux. It seemed that mSOD1 fibroblasts were in a “fasted-like-state” with limited FAO fueled respiratory flux compared to controls, either as a pathogenic consequence of the SOD1 mutation, or a physiological consequence of upregulated glycolysis. Similar “starvation” phenotypes have been previously reported in both SOD1 and TDP-43 transgenic models and correspond closely to the hypolipidemic state previously observed in ALS patients.^142^ This parallel upregulation of glycolysis might be either a compensatory response to the loss of mitochondrial ATP synthesis, or, on the contrary, an effect of the TDP-43 and SOD1 mutations which further drives mitochondrial uncoupling and limits ATP production via the ETC. A possible explanation to account for these metabolic modifications could implicate the canonical WNT/β-catenin pathway, an apparently crucial factor in the progression and development of many neurodegenerative diseases.^143^ Previous research reported upregulation of WNT/β-catenin signaling in the motor neurons of SOD-1-ALS transgenic models,^144^ which, in return, may activate pyruvate dehydrogenase kinase-1 and consequently hinder the conversion of pyruvate into acetyl-CoA and hampers its entry into the tricarboxylic acid (TCA) cycle. Overexpression of WNT/β-catenin pathway also activates monocarboxylate lactate transporter-1, which facilitates the transport of lactate into the extracellular environment.^145^ Moreover, upregulating the activity of the peroxisome proliferator-activated receptor gamma (PPAR gamma), a repressor of the beta-catenin pathway, exerted neuroprotective effects in TDP-43 and FUS *Drosophila* models of ALS.^146^ This possible association between the WNT/β-catenin signaling pathway and metabolic changes is supported by evidence from experiments on the NSC-34 cell line, in which expression of mutant SOD1G93A led to increased aerobic glycolysis as a consequence of pyruvate dehydrogenase kinase 1 activation.^147^ However, in the NSC-34 study, the increase in glucose metabolism impaired the ability of the cells to adapt to stress and promoted cellular death, making it difficult to conclude whether the increase in glycolytic flux is neuroprotective or has detrimental effects on motor neurons. In line with the effects of SOD1 on FAO observed in our laboratory, recent experiments in a TDP-43 *Drosophila* model of ALS revealed that TDP-43 pathology alters the transcription of crucial components of the carnitine shuttle, leading to a significant reduction in lipid beta oxidation.^148^ Although it is unclear what underlines this distinctive malign activity of TDP-43, dietary supplementation with medium-chain fatty acids, which independently cross the mitochondrial membrane, mitigated the carnitine deficit and rescued motor neuron survival.

Contradicting the evidence that glycolysis might increase in ALS in an attempt to counterbalance the loss of mitochondrial contribution to energy production, gene expression profiling in fibroblasts from sALS suggested a dysregulation of glycolysis and mitochondrial oxygen consumption in the pathogenesis of the disease, as a result of reduced expression of insulin signaling receptors and of key enzymes of the TCA cycle.^149^ The authors propose that, as glucose oxidation decreases and mitochondrial defects progress, cells switch the energy substrate to fatty acids, a possibility supported by the upregulation of genes responsible for fatty acid transport and oxidation as shown by microarray analysis. Moreover, Szelechowski (2018) suggest that both glycolysis and FAO are decreased in fibroblasts from SOD1-ALS patients, whereas SOD1G93A motor neurons report increased activity in these two energy-producing pathways, with a larger preference for FAO.^150^ The study supports the existence of tissue-specific alterations in ALS pathophysiology and suggests that changes in mitochondrial morphology and mitochondrial bioenergetics accommodate a metabolic switch from glucose toward FAO and ketogenesis. This hypothesis is supported by dietary intervention studies which highlighted the positive effects on disease onset and survival of high fat diets in TDP-43 and SOD1 mice,^151^ as well as ALS patients.^95,152^ To possibly explain the metabolic preference for FAO in the context of SOD1 pathology, Szelechowski (2018) highlights previous findings which show that abnormal SOD1 binds to the mitochondrial outer membrane and inhibits conductance through voltage dependent anion channels (VDACs), thereby leading to bioenergetic blockade.^153^ Considering that fatty acids have the ability to diffuse through the mitochondrial membrane, FAO could represent a more sustainable energy-transducing pathway which by-passes this energetic impairment. However, even though previous studies have shown that TDP-43 interacts with VDACs as well, influencing the mitochondrial dynamics,^154^ it is still unknown whether it leads to similar downstream changes as seen in SOD1 cases, and a comprehensive characterization of the context in which these metabolic changes occur is yet to be put forward.

When discussing energy balance in the nervous system, it is important to note that neurons do not function as isolated units, and their unique energy capacity makes them dependable on surrounding cells like astrocytes and oligodendrocytes for metabolic support. ^134,155^ During intense synaptic activity astrocytes take up glucose and metabolize it into pyruvate, which gets further converted to lactate. It has been postulated that lactate is then delivered to neurons via the astrocyte-neuron lactate shuttle, where it is converted back to pyruvate and enters the TCA cycle to feed into oxidative phosphorylation.^156^ It is well established now that ALS has a non-cell-autonomous character where astrocytes are a particularly important target of disease pathogenicity.^157^ Any impairments in metabolic pathways within astrocytes or reductions in their ability to optimally deliver the necessary substrates to neurons could profoundly affect neuronal homeostasis and contribute to neurotoxicity and cell death. In line with this, the monocarboxylate transporter 1 (MCT1), an important modulator of lactate transport, has been shown to be downregulated in TDP-43 astrocytes cultures^158^ and in spinal cord oligodendrocytes from SOD1 transgenic mice,^159^ whereas MCT4 measured decreased expression in SOD1 astroglia.^159^ In their recent study, Velebit et al. (2020) suggest that astrocytes which exhibit TDP-43 inclusions suffer alteration in aerobic glycolysis and accumulation of lipid droplets, which consequently perturb astroglia metabolism and impair the trophic support of neurons.^158^ Moreover, we have recently reported an altered metabolic profile in induced astrocytes from C9ALS patients, where defects in nucleoside, glycogen, pyruvate and fructose metabolism corresponded to a decrease in metabolic flexibility.^137,160^ It appears that *C9orf72* induced astrocytes have a decreased capacity to mobilize glycogen, an aspect that could reduce glucose levels and consequently minimize ATP production in times of bioenergetic stress.^137^

**Figure 6.**
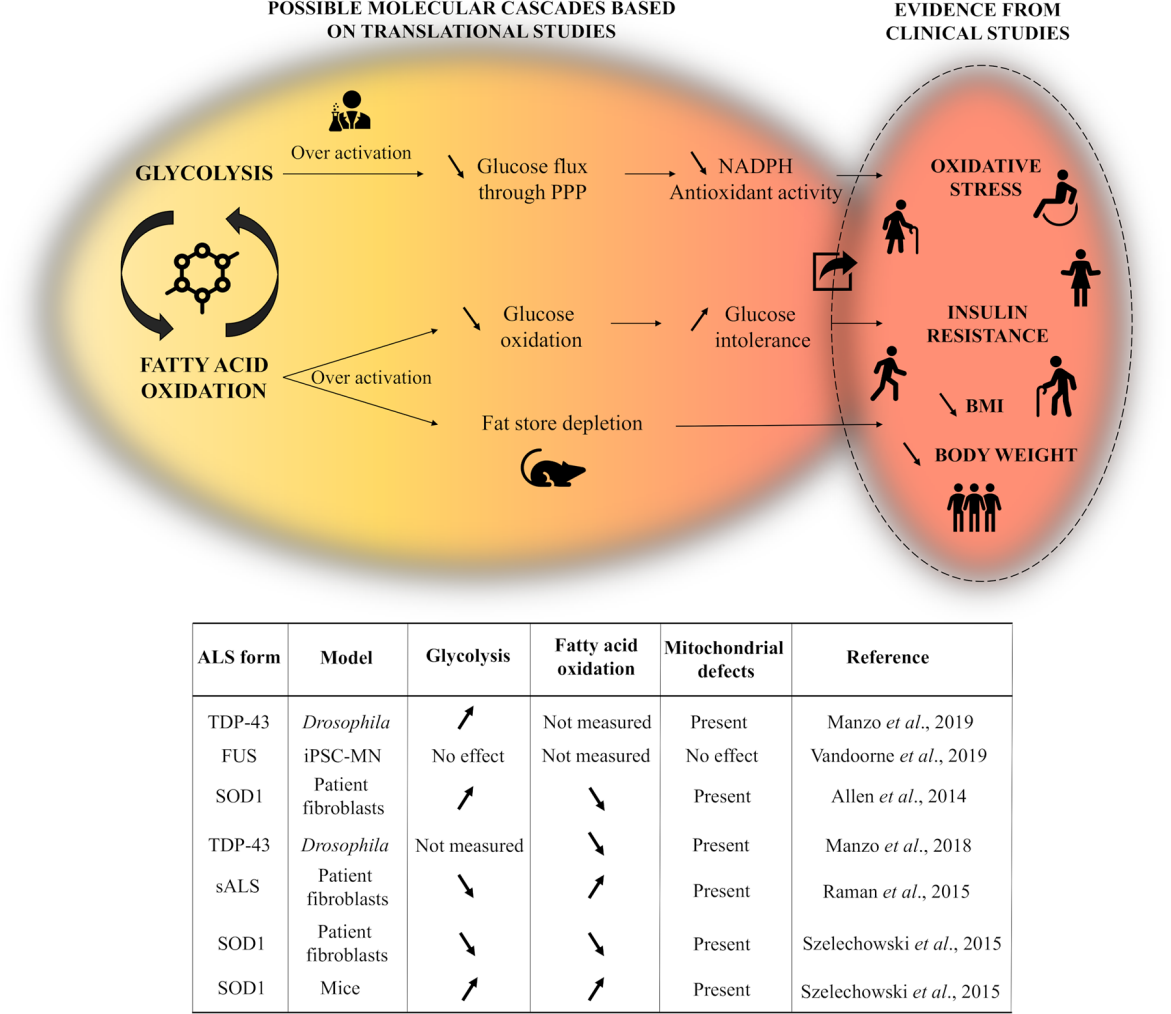
Graphic representation of the possible effects of upregulating glycolysis or fatty acid oxidation, and their correlation with clinical data (upper figure). Table summarizing the findings of the aforementioned studies on the contribution of glycolysis and fatty acid oxidation to restoring metabolic homeostasis (lower table).

Even though the aforementioned studies investigate two separate metabolic pathways, FAO and glycolysis, in genetically distinct models of ALS, their findings emphasize the crucial role of metabolic flexibility in ALS, and capture the attempt of the cells to mitigate metabolic stress in order to restore energy homeostasis. Both pathways have the capacity to modulate the activity of the other, but considering the complex downstream signaling cascades influenced by these major energy generating-systems, it remains an open question whether increasing the activity in one to the detriment of the other is beneficial in the long term or not (Figure 6). The possible increase in FAO discussed above could potentially further facilitate the inhibition of glucose oxidation, leading to glucose intolerance and insulin resistance, as previously reported in ALS patients.^161^ Moreover, patients with ALS often report dysphagia and a consequential reduction in food intake,^162^ which, coupled with a possible dependence on FAO to meet the energy needs, may eventually lead to a depletion of fat stores. Indeed, a decline in BMI and loss of body weight is a common aspect in ALS patients, which correlates with a more rapid progression of the disease.^163^ On the other hand, increasing glycolysis in cortical neurons could reduce the flux of glucose through the pentose phosphate pathway, consequentially diminishing the antioxidative contribution of NADPH (Figure 6). Nevertheless, it is also very important to note that the high variability of the findings discussed in the section above, and by extrapolation, in the neuroscience literature at large, must be considered in the context of research methodologies and translational limitations. In a preclinical context, the biological process of interest is manipulated by a scientist in an animal model before the results are correlated with data from clinical studies. This approach allows for variability and the possibility of errors being introduced as each methodological step. Such limitations must be considered when analysis and integrating novel findings with the preexisting dogma.

It is still soon to conclude what exactly are the shifts in metabolic pathways that ALS may trigger, and to declare with certainty whether they are beneficial compensatory mechanisms or a result of ALS-associated mutagenesis. However, it is indisputably clear that neurons and glial cells are trying to respond to the metabolic defects accompanying disease progression, and future research is needed to devote to understanding these responses as they could represent potential targets for therapeutic strategies.

## Where does AMPK signaling sit in the context of TDP43-ALS pathogenesis?

AMP-activated protein kinase (AMPK) is a master regulator of energy homeostasis which is gaining increasing attention in the context of metabolic dysfunction in ALS. AMPK signaling is a crucial factor in detecting the balance between energy supply and demand, having a pivotal role in many intracellular regulatory pathways that range from energy metabolism to cellular stress.^164^ In a pathological state, the AMPK pathway is activated to reduce energy expenditure and to facilitate glucose utilization when ATP levels become critical, either due to its production being compromised or its degradation being abnormally enhanced.^164^ Considering that energy homeostasis becomes impaired in ALS, due to abnormal activity in any of the energy-generating pathways discussed above, AMPK signaling might also be consequently impeded and may contribute to disease pathogenesis. Indeed, recent studies suggest that AMPK cellular activity is dysregulated in the context of ALS pathology, but evidence to support the signaling mechanisms involved is unfortunately insufficient and limited to TDP-43 and SOD1 pathologies.

Previous research has shown that upregulation of the AMPK pathway facilitates the mislocalization of TDP-43 in NSC-34 cells via a positive feedback mechanism in which TDP-43 plays the central role.^165^ It appears that cellular stress and elevated levels of ROS activate the AMPK pathway, which, possibly due to its role in nucleocytoplasmic transport,^166^ mediates the mislocalization of TDP-43. In turn, TDP-43 contributes to additional cellular oxidative stress which further enhances AMPK signaling.^165^ Moreover, increased AMPK activity also impaired the normal subcellular distribution of FUS, highlighting the critical role of AMPK-mediated pathogenicity in ALS.^167^ Supporting these results, pharmacological or genetic downregulation of the AMPK pathway resulted in decreased TDP-43 mislocalization and delayed neurodegeneration progression in NSC-34 cells or *C. elegans* models of disease.^165,168^ However, Perera et al. (2014) showed contradictory findings, suggesting that mutant TDP-43 inhibits AMPK activity by positively acting on AMPK phosphatase and protein phosphatase 2A, two critical negative regulators of AMPK.^169^ As the later experiments were performed on a transgenic model of TDP-43-associated ALS, these contrasting results might be attributed either to different methodologies or to differences in the biological systems used or mutation investigated. Interestingly, in Perera’s study (2014), the SOD1 cohort reported a sharp increase in AMPK phosphorylation at disease onset. These contrasting results between the TDP-43 group and the SOD1 mice might reflect distinct pathogenic mechanisms or important differences in energy metabolism between these highly prevalent genetic causes of ALS.

Indeed, when it comes to SOD1 pathology, the evidence so far converges toward a consensus which suggests the presence of enhanced activation of the AMPK energy sensor in both cell lines and animal models of disease,^164,168^ as well as ALS patients.^165^ Surprisingly, however, stimulation of AMPK signaling in SOD1 models has led to conflicting results. Metformin-mediated activation of AMPK accelerated symptom onset and disease progression in SODI mice,^170^ whereas AMPK activation as a result of resveratrol administration showed beneficial effects on motor function and lifespan.^171^ A possible explanation to support the later finding comes from previous studies which showed that resveratrol acts on the NAD-dependent deacetylase sirtuin-1 to activate the AMPK in a dose dependent manner,^172^ which further deacetylates and positively regulates PGC-1α.^173^ As PGC-1α is a co-activator of the nuclear respiratory factors and crucial regulator of mitochondrial biogenesis,^174^ AMPK may consequently improve mitochondrial function in skeletal muscles. However, it is important to note the characteristic differences between cellular respiration in skeletal muscles and neuronal metabolism, and to interpret the aforementioned findings keeping in mind that AMPK modulation might have different effects in oxidative muscle fibers compared to glycolytic neuronal cells. To support this thought, no changes in PGC-1α were observed in spinal cord neuron cultures as a result of AMPK activity modulation, further emphasizing that any cellular changes observed might differ between cell-types or could be lost in whole tissue preparations.^168^

The detrimental effects of increased AMPK activity satisfy the expectations one might have when considering the possible downstream mechanisms of AMPK upregulation. Stimulating AMPK activity, which at the cellular level promotes glucose uptake and positively regulates FAO, could provide additional damage since both these pathways might be compromised in ALS, as discussed in the section above. In the same way, even though AMPK activation might have a positive contribution to turning on catabolic pathway in a healthy biological system, constant upregulation of AMPK signaling might become detrimental in the context of ALS pathology, in which high metabolic demands are inflicted on neurons, energy stores are depleted and critical anabolic cascades are impaired. This possibly dual character of AMPK is supported by Coughlan et al. (2015)^175^ who highlights the benefits of a “preconditioning” approach when modulating AMPK function, suggesting that, while chronic activation of AMPK might be detrimental, subthreshold stimulation might increase tolerance for a potentially more robust future stress. Whether AMPK upregulation is beneficial or not, and how ALS pathogenesis affects this energy sensor remain two important open questions that need to be answered, but which encourage the existence of impaired energy homeostasis in ALS.

## Metabolomics biomarkers in ALS

The well recognized presence of metabolic defects in the pathogenesis of ALS, along with the increasingly growing notion that ALS patients exhibit a hypermetabolic state^1,8^ has brought the field of metabolomic research forward in the study of ALS, aiming to identify ALS-specific metabolites that could further represent physiological biomarkers of disease and which could possibly contour a metabolic signature for ALS.

So far, metabolomics analysis has proven effective in identifying particular metabolic pathways affected in the disease progression, pathways which could then be targeted and compensated through therapeutic manipulation.^176^ Although metabolomic screening has been mainly focused on patients bearing mutations in SOD1 to the detriment of other genetic causes of ALS, in vitro and in vivo experiments shed some light on the association between TDP-43 pathology and metabolic alterations. A recent study in a *Drosophila* model of TDP-43-associated ALS revealed a significant increase in long-chain fatty acid carnitine conjugates such as myristoylcarnitine, palmitoylcarnitine, oleoylcarnitine and linoleoylcarnitine, in the context of both mutant and wild-type TDP-43 overexpression.^148^ In line with these observations, an important product of lipid beta oxidation, beta-hydroxybutyrate, along with the level of carnitine, a critical modulator of fatty acid transport across the mitochondrial membrane, were decreased in both experimental groups. Additional quantitative polymerase chain reaction (qPCR) analysis suggested that the expression of major units of the carnitine shuttle pathway was affected at the transcriptional level, pointing toward major defects in the mitochondrial carnitine shuttle system in the context of TDP-43 pathology. These findings support data from clinical trials where low levels of carnitine have been reported in the blood and CSF of ALS patients,^177,178^ possibly correlating with the presence of amyotrophy, a primary ALS symptom. Similar alterations in lipid profiles have been previously reported in a cellular model of TDP-43, with polyunsaturated fatty acids (PUFA) and glycerophospholipids being particularly affected.^179^ However, these findings were recorded in the context of wild-type TDP-43 overexpression, providing evidence for the toxic role of endogenous TDP-43, which will be discussed in detail below. Interestingly, comparable decreases in glycerophospholipids levels and matching alterations in PUFA concentrations had been formerly shown in a SOD1 transgenic model^180^ and ALS patients respectively.^181^ Moreover, lipidomics analysis on the ALS mouse model bearing the SOD1-G93A mutation distinguished altered phosphatidylcholine profiles in the cerebral cortex,^182,183^ possibly indicating a common pathogenic mechanism between TDP-43 and SOD1 toxicity. Similarly, high levels of free fatty acids and liquid droplets confirmed a defective lipid metabolism in iPSC-derived motor neurons from *C9orf72* ALS patients,^184^ but it remains to be confirmed whether TDP-43 pathology could possess any active contribution to these metabolic defects.

Nevertheless, metabolomic analysis suggests that the detrimental effects of TDP-43 are not restricted to lipid metabolism, but extends to other energy producing pathways as well, encompassing metabolic cascades like glycolysis in their pathogenicity. A recently published study performed in the same ALS-TDP-43 *Drosophila* model revealed increased levels of glycolytic metabolites such as phosphoenolpyruvate, pyruvate and sedoheptulose-7-phosphate in whole larvae, possibly indicating a pronounced glycolytic input into the pentose phosphate pathway as an attempt to mitigate oxidative stress through increased NADPH production.^138^ These results are consistent with elevated plasma pyruvate levels reported in ALS patients.^185^ Further analysis on patients derived iPSC motor neurons and transcriptional profiling of ALS spinal cord suggested a significant upregulation of phosphofructokinase-1 levels, confirming an increased glycolytic flux in the context of TDP-43 pathology.^138^ Similar changes in metabolites profile were reported in NSC-34 cells expressing mutant SOD1-G93A as well, where increased lactate, alanine and formate levels in the medium were indicative of pronounced glycolysis.^147^ Interestingly, this imbalance in ATP homeostasis could explain recent findings from sporadic ALS patients’ fibroblasts which showed alterations in purine and pyrimidine metabolism. The authors suggested that a disturbance in ATP production or abnormally elevated ATP consumption rates could lead to significant changes in the serum profiles of purines, since these compounds are directly connected to ATP synthesis. Similarly, recent work from our laboratory discovered defects in purine metabolism, specifically adenosine, in astrocytes derived from sporadic and *C9orf72* ALS cases due to loss of adenosine deaminase.^137,160^ It remains to be seen whether purine/pyrimidine metabolism is affected in TDP43 cases. However, the fact that nucleoside defects are observed in C9orf72 and sporadic ALS cases of disease, indicate a common pathogenic mechanism of disease that warrants further research including the possibility of targeted nutritional supplementation.

The studies discussed above provide evidence for the crucial contribution of metabotype information to identifying biochemical pathways that are affected in ALS patients. Furthermore, by comparing the metabolic state at baseline with that measured after the administration of a proposed drug or therapy, scientists could improve their knowledge regarding treatment outcomes and better assess the drugresponse.^186^

## TDP-43 mutations—a requisite of TDP-43 pathology? The contribution of wild-type TDP-43 to neurodegeneration

Although the involvement of TDP-43 mutations in ALS pathogenesis cannot be contested, just a considerably small percentage of ALS cases are familial, and many other neurodegenerative disorders report the existence of TDP-43 pathology in the absence of known TARDBP mutations.^187,188^ We have so far established the critical roles TDP-43 possess during the shift of homeostatic balance into progressive neurodegeneration, but we have not yet addressed the question of whether there is a difference in functional activity between wild-type and mutant TDP-43, and whether mutations in the TARDBP gene are considerably more pathogenic than the wild-type form.

Genetic upregulation of wild-type TDP-43 showed rapid formation of cytoplasmic aggregates in bacteria and yeast, along with a sharp decrease in growth rate, changes in morphology and even cell death in yeast.^189,190^ Earlier studies in mouse models have reported that overexpression of wild-type human TDP-43 facilitated the accumulation of TDP-43 in nuclear and cytoplasmic aggregates, which further resulted in cortical and spinal neuronal degeneration accompanied by spastic paralysis.^37^ TDP-43 phosphorylation and aggregation in the spinal cord was again described in another transgenic mouse model overexpressing human TDP-43,^126^ along with a dose-dependent downregulation of mouse TDP-43. Interestingly, abnormal accumulation of mitochondria within motor neurons was also measured by Xu et al. (2010), indicating possible deficits in axonal transport machinery in the human TDP-43 cohort. Moreover, the findings reported an increased expression of two crucial components of mitochondrial fission machinery, dynamin-like protein and mitochondrial fusion 1 protein (DNM1L), which was accompanied by a downregulation of mitofusin 1 (MFN1), a major player in mitochondrial fusion. In the light of these results, it may be possible that the post-translational modifications of TDP-43 come prior to mitochondrial clustering in disease progression. However, the TDP-43 phosphorylated inclusions were primarily located in the spinal cord, and more sparsely in the brain, possibly suggesting that the effects of human TDP-43 expression depend on the anatomic region and the biochemical context in which it is expressed. More recent studies compared the independent expression of human wild type TDP-43 (TDP-43 wt) and the expression of Q331K mutant (TDP-43 Q331K) against the co-expression of wild-type and mutant form (TDP-43 WTxQ331K).^191^ Surprisingly, the TDP-43 WT group did not show any clinical or pathological phenotype, whereas the mutant cohort reported cytoplasmic accumulation of TDP-43 along with mild neuronal loss and glial activation in the spinal cord and the motor cortex. When analyzing the mixed group, however, an extremely aggressive motor phenotype was reported, with significant reactive gliosis and neurodegeneration in the cortex and spinal cord, and prominent TDP-43, p62 and ubiquitin cytoplasmic inclusions. Taken together, the results of the study indicate that, while the expression of human TDP-43 is not lethal on its own, its destructive effects could be profoundly accentuated by the presence of a pathogenic mutation, and that cytoplasmic accumulation of TDP-43 is sufficient to lead to neurodegeneration, without the contribution of loss of nuclear function. On the other hand, it may be possible that the wild-type TDP-43 contributes in a dose-dependent manner to neuronal toxicity by reinforcing the aggressive pathogenic activity of mutant TDP-43. This paradigm is confirmed and reinforced by further studies in mammalian models, which showed sparse aggregation of TDP-43 and surprisingly high levels of cytotoxicity, following overexpression of wild-type TDP-43.^192,36^ In an elegant study, Capitiniet al. (2014)^193^ used purified wild-type TDP-43 aggregates derived from *Escherichia coli* and transfected them into SH-SY5Y cells, to allow a direct analysis of TDP-43 aggregation alone. The results showed elevated levels of ROS and caspase-3 activation, along with a consequential significant decrease in viability. Interestingly, TDP-43 was not mislocalized from the nucleus and did not aggregated into cytoplasmic inclusions, indicating that wild-type TDP-43, when overexpressed, is sufficient to induce cellular toxicity and ALS-like phenotypes, but, contrarily to TDP-43 mutants, the wild-type allele retains its nuclear localization and rarely aggregates. This hypothesis still needs to be confirmed as, so far, the research investigating the pathogenicity of wild-type TDP-43 shows conflicting results.

As recognized by previous studies, ALS-associated TDP-43 mutations possess higher stability and show increased half-life compared to the wild-type form.^194,195^ Moreover, disease-linked mutants are more rapidly turned over through the ubiquitin/proteasome system (UPS) system than wild-type TDP-43,^196^ making it likely that such modifications are the driving factors that alter the processing and translation of the TDP-43’s mRNAs and facilitate its mislocalization and precipitation. Taken together, the studies discussed above advocate a possible pathogenic contribution of wild-type TDP-43 to disease progression in ALS. Whether the other genetic drivers of ALS have a similar function in their wild-type form goes beyond the scope of this review, but, considering its potential implications for research methodologies, such possibility should be further investigated in the future. Experimental control groups are crucial for validating the significance of the results, and selecting the right controls is a challenging task that can deeply influence the outcome of data analysis. Hence, it is crucial to consider the potential pathogenicity of WT proteins when designing our chosen research methods in order to rule out any contribution of its activity.

## Conclusions

Considering the solid circumstantial evidence discussed above, one can undoubtedly state that TDP-43 contributes majorly to metabolic dysfunction and energy defects in ALS pathogenesis. From changes in mitochondrial dynamics to alterations in metabolic profiles, TDP-43 pathology appears to integrate a wide spectrum of structural, morphological and functional defects. However, it is yet too early to conclude which is the precise origin of TDP-43 pathogenic signaling and how it differs from other ALS-associated genetic risks such as SOD1, FUS or *C9orf72*, as there are still major gaps in our current understanding of the underlying disease mechanisms. At the same time, it remains an open question of how different genetic causes of ALS can have opposing pathogenic activities regarding some aspect of ALS pathogenesis, while still eventually converging toward the same pathological outcome on motor neurons. Moreover, it is important to consider that, as ALS is a disease with a major detrimental effect on visceral and skeletal muscle, metabolic changes occurring at this level could provide great insights into what may be happening upstream, at higher cortical levels. In a recently published study, the levels of sarcolipin, an important regulator of SERCA activity in muscle fibers, appeared to be upregulated in SOD1 transgenic mice,^197^ triggering a mitochondrial reprogramming event which determines a transition of skeletal muscle for glycolytic to oxidative metabolism.^198^ Such modifications correlate well with some of the evidence discussed above, supporting the existence of metabolic dysfunction and early mitochondrial failure in ALS pathogenesis. However, future work is needed for an in-depth characterization of the systemic abnormalities associated with ALS, and an exhaustive understanding of these metabolic changes is crucial in terms of finding potential therapeutic strategies.
